# In Vitro and In Silico Studies of Functionalized Polyurethane Surfaces toward Understanding Biologically Relevant Interactions

**DOI:** 10.1021/acsbiomaterials.3c01367

**Published:** 2023-11-01

**Authors:** Paulina Chytrosz-Wrobel, Monika Golda-Cepa, Kamil Drozdz, Jakub Rysz, Piotr Kubisiak, Waldemar Kulig, Monika Brzychczy-Wloch, Lukasz Cwiklik, Andrzej Kotarba

**Affiliations:** † Faculty of Chemistry, Jagiellonian University in Krakow, Gronostajowa 2, 30-387 Krakow, Poland; ‡ Department of Molecular Medical Microbiology, Chair of Microbiology, Faculty of Medicine, Jagiellonian University Medical College, Czysta 18, 31-121 Krakow, Poland; § Faculty of Physics Astronomy and Applied Computer Science, Jagiellonian University, Lojasiewicza 11, 30-348 Krakow, Poland; ∥ Department of Physics, University of Helsinki, P.O. Box 64, FI-00014 Helsinki, Finland; ⊥ J. Heyrovský Institute of Physical Chemistry, Czech Academy of Sciences, Dolejškova 3, 18223 Prague, Czech Republic

**Keywords:** polyurethane, oxygen plasma, biocompatibility, bacteria adhesion, molecular dynamics

## Abstract

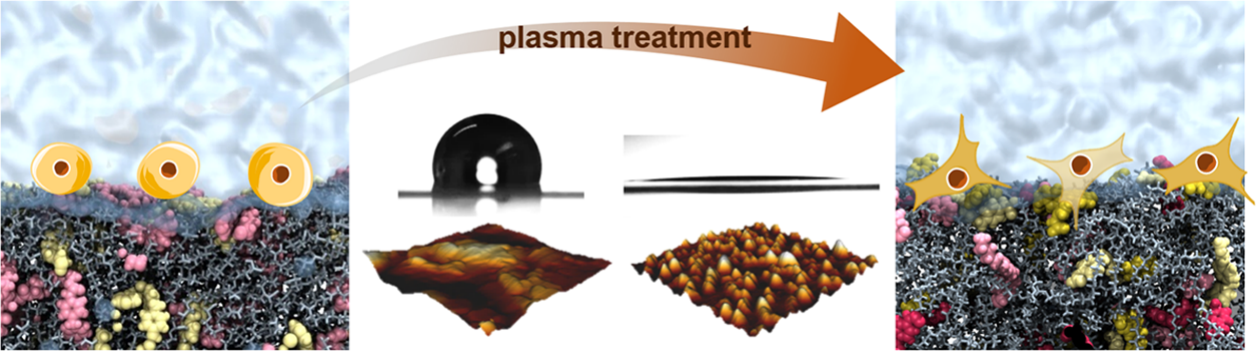

The solid–aqueous boundary formed upon biomaterial implantation provides a playground for most biochemical reactions and physiological processes involved in implant–host interactions. Therefore, for biomaterial development, optimization, and application, it is essential to understand the biomaterial–water interface in depth. In this study, oxygen plasma-functionalized polyurethane surfaces that can be successfully utilized in contact with the tissue of the respiratory system were prepared and investigated. Through experiments, the influence of plasma treatment on the physicochemical properties of polyurethane was investigated by atomic force microscopy, attenuated total reflection infrared spectroscopy, differential thermal analysis, X-ray photoelectron spectroscopy, secondary ion mass spectrometry, and contact angle measurements, supplemented with biological tests using the A549 cell line and two bacteria strains (*Staphylococcus aureus* and *Pseudomonas aeruginosa*). The molecular interpretation of the experimental findings was achieved by molecular dynamics simulations employing newly developed, fully atomistic models of unmodified and plasma-functionalized polyurethane materials to characterize the polyurethane–water interfaces at the nanoscale in detail. The experimentally obtained polar and dispersive surface free energies were consistent with the calculated free energies, verifying the adequacy of the developed models. A 20% substitution of the polymeric chain termini by their oxidized variants was observed in the experimentally obtained plasma-modified polyurethane surface, indicating the surface saturation with oxygen–containing functional groups.

## 1 Introduction

Polyurethanes make up a large family of polymers widely employed in medical devices, and they have one common characteristic: the presence of urethane linkages along their large molecular chains. The urethane linkages are formed by the reaction of isocyanates (−NCO) and alcohols (−OH) and generally account for only a small fraction of the polymer chain.^1−8^ Owing to their versatility caused by their chemical composition, polyurethanes are currently the best-performing biomedical-grade elastomers. The controlled adjustment of the properties of polyurethanes relies heavily on the macroglycol portions of the polymer chain and, accordingly, on the choice of macroglycol, e.g., polyesters, polyethers, or polycarbonates. Due to the different chemical reactivities of the specific functional groups, polyesters are hydrolytically labile (hence their prevalence in biodegradable applications), whereas polyethers are hydrolytically stable (relevant for long-term biomaterials such as artificial blood vessels). The combination of polyesters and polyethers can be exploited to achieve the desired degradation rate in various applications. Generally, polyester components offer excellent mechanical properties, whereas polyethers provide flexibility and hydrophilicity to copolymers.^2,9,10^ Thus, polyurethanes can be rigid, semirigid, or flexible with excellent biocompatibility, outstanding hydrolytic stability, superior abrasion resistance, excellent physical strength, and high flexural endurance.^11,12^ These features enable their diverse gamut of bioapplications as components of pacemakers, urinary catheters, vascular grafts, heart-assist balloon pumps, artificial heart bladders, and wound dressings.^11,13−17^

Understanding solid–water interfaces in biomaterials science is essential for apprehending their functions since these interfaces provide a natural playground for most biochemical reactions and physiological processes involved in host–implant interactions.^18,19^ Therefore, the surface functionalization of polymeric materials via the generation of functional groups is currently at the forefront of biomaterials research. This is also true for polyurethanes since their bulk properties are sufficient to enable their use in carrying out a desired tissue function of the body, e.g., bone or vein functions. However, the surfaces of the raw materials are generally not compatible with the adjacent tissue. Although their mechanical properties, wettability, and biocompatibility have been investigated, other important materials’ features have to be tested, e.g., their susceptibility to bacterial adhesion. This is particularly important because immediately after the implantation, the so-called “race for the surface” occurs between the eukaryotic cells and the bacteria cells.^20^

Recently, cold plasmas have proved effective for material functionalization, owing to their good performance at close-to-ambient temperatures.^21−23^ The major advantage of plasma treatment, compared with the “wet chemistry” method, is its versatility, as it is not limited to a specific polyurethane structure. However, the treatment parameters need to be adjusted individually for each material, considering, for example, its chemical composition, crystallinity, and thickness. The advantage of plasma treatment lies in the precise control of the functionalization degree, achieved by parameter adjustment (the type of feed gas: oxidative, reductive, inert; gas partial pressure; the power of the plasma generator and exposure time).

As polymers are insulating materials, the introduced functional groups form stable surface dipoles, which lead to the formation of an electrical double layer. The role of such accumulated surface charges is particularly pronounced in the polymer–protein interactions. Moreover, prolonged plasma treatment remodels the surface topography, which is mostly observed for semicrystalline polymers. This is because the amorphous regions are preferentially etched, whereas the crystalline regions are more plasma-resistant.^24,25^ Notably, only by the fine adjustment of the plasma-treatment parameters will the functionalization be confined to the shallow surface layer (∼10 nm) of the polymer,^26^ providing homogeneously distributed functionalities throughout the surface. This is strongly desired in the context of biomaterials where the surface and biological properties need to be modified while preserving the bulk and mechanical properties.^27^

Although polyurethanes are widely employed in the biomedical field and have been extensively studied, comprehensive molecular-level insights into their properties remain unavailable. More importantly, the polyurethane material surfaces and their interactions with water on the nanoscale are poorly understood. Such molecular-level insights can be provided by molecular dynamics (MD) simulations. The existing MD simulation studies of polyurethane are limited to relatively short lengths- and time scales in the range of a few nanoseconds in atomistic simulations or a few tens of nanoseconds in coarse-grained models.^28−31^ Furthermore, these studies mostly focus on the properties of the bulk material. Here, we aim to describe the polyurethane–water interface in relation to the material’s biocompatibility. To this end, we introduced and developed new, realistic, and fully atomistic models of the polyurethane material and employed them to characterize, in detail, the interfacial properties of the material at the nanoscale. Notably, the models are compatible with existing force fields for biomolecules, enabling their future application in exploring polymer–biomolecule interactions. Moreover, in this study, we exploit the recent advances in high-performance computing and simulate the atomistic polyurethane models at the microsecond times- and tens of nanometers length scales.

We investigated oxygen plasma-functionalized biocompatible polyurethanes that can be successfully utilized in contact with the tissue of the respiratory system. We performed a series of experiments to optimize the plasma-treatment parameters and test the effect of the treatment on the physicochemical properties (e.g., chemical composition and wettability). The experimental studies were supplemented with atomistic MD simulations of the newly developed polyurethane model to understand the fundamental processes at the polyurethane–water interface. Finally, we tested the adhesion of bacterial and human epithelial cells at the surface of functionalized polyurethane, confirmed its biocompatibility, and discussed it in the context of biomaterial-centered infections (BCIs).

## 2 Materials and Methods

### 2.1 Material Preparation and Functionalization

Medical-grade polyurethane (Aromatic Polyether Polyurethane, 82 Shore A) films were purchased from American Polyfilm, Inc. The thickness of the polyurethane layer used in the experiments was 100 μm. Before each experiment, polyurethane foils were washed with 2-propanol (Avantor) and air-dried. To generate oxygen-containing functional groups and nanotopography, the polyurethane samples were treated by oxygen plasma (FEMTO system, Diener Electronics) at a controlled oxygen partial pressure of 0.14 mbar (Oxygen SIAD Poland 6.0 pure 99,9999%), plasma generator power of 50 W, and sample exposure time of 1 or 10 min.

### 2.2 Physicochemical Characterization of the Polyurethane Films

#### 2.2.1 Attenuated Total Reflection Infrared (ATR-IR) Spectroscopy

Modified samples of polyurethane were examined using a Nicolet 6700 system (Thermo Scientific) with a diamond crystal. Each of the studied samples was scanned 64 times in the range of 600–4000 cm^–1^.

#### 2.2.2 Differential Scanning Calorimetry (DSC)

The oxygen-plasma modification degree (surface and bulk) was verified using a DSC 821e Mettler Toledo instrument. The analysis was conducted in a temperature range of −100 to 300 °C, at a heating rate of 10 °C/min, in Ar flow.

#### 2.2.3 X-ray Photoelectron Spectroscopy (XPS)

The polyurethane samples were analyzed by XPS to identify the surface functional groups formed upon plasma treatment. The measurements were conducted in an ultrahigh vacuum chamber (vacuum level >5 × 10^–9^ mbar) using an SES R4000 analyzer (Gammadata Scienta), with a monochromatic Al Kα X-ray source (1486.6 eV) at 250 W (pass energy: 100 eV) for the survey and narrow scans. The electron binding energy of the C 1s peak was calibrated at 285 eV. The obtained XPS spectra were analyzed using the Casa-XPS 2.3.15 software.

#### 2.2.4 Secondary Ion Mass Spectrometry (SIMS)

SIMS was employed as a complementary method for investigating the polyurethane surface composition. Static mass spectra were acquired using a TOFSIMS 5 system equipped with a time-of-flight MS analyzer (IONTOF GmbH, Germany). The changes in the polyurethane surface composition were monitored using the mass spectra of the secondary positive ions induced by the Bi^3+^ (30 keV, 0.7 pA) primary beam rastered over an area of 250 μm × 250 μm. For statistics, data from five randomly selected spots were collected for each sample. The obtained data were analyzed and interpreted using the dedicated SurfaceLab 7.0 software.

#### 2.2.5 Atomic Force Microscopy (AFM) and Image Processing

For a more detailed characterization of the surface topography of the polyurethane samples, including the *Z*-axis, AFM in contact mode was performed. The measurements were performed using a JPK NanoWizard 4 XP system with an Olympus optical microscope having an active vibration isolation platform (Accurion i4) and acoustic enclosure. The AFM cantilever tips of the Bruker instrument (TESPG-V2) were operated in contact mode with a Si material doped with an Sb tip at a typical spring constant of ∼42 N/m and resonance frequency of ∼320 kHz. Surface scanning was performed perpendicular to the axis of the cantilever with an applied frequency of 0.5 Hz, with the experimental scan area being 400 nm × 400 nm. The topographic data were processed using the JPK Data Processing software, and the roughness was parametrized by the root-mean-square (RMS).

#### 2.2.6 Water Contact Angle Measurements and Surface Free Energy (SFE) Calculations

The changes in the wettability of the polyurethane surfaces were monitored by water contact angle measurements using a Surftens Universal goniometer (OEG GmbH). The static contact angles were calculated by using dedicated image processing software (Surftens 4.3, in the automatic mode). For each sample, three independent series were investigated, and the mean value was obtained for five 1.5-μL water drops. The SFE calculations were performed using the Owens–Wendt method, which requires two liquids with different polar components, as described elsewhere.^32^

### 2.3 Biological Tests

The biocompatibility tests of the unmodified and oxygen-plasma-modified polyurethane samples were performed using the adenocarcinoma human alveolar basal epithelial cell line A549. The cells were grown in Dulbecco’s Modified Eagle’s Medium (DMEM) (with l-glutamine, 10% FBS, ZellShield) at 37°C with 5% CO_2_. Thereafter, the cells were seeded on the polyurethane samples in 24-well plates with a density of 5000 cells/cm^2^ and incubated for 24 h with the polyurethane samples. Subsequently, the cells were washed with phosphate-buffered saline (PBS), fixed in 4% paraformaldehyde, and made permeable in TritonX100. To stain F-actin and the cell nuclei, Alexa Fluor 488 dye and DAPI (Molecular Probes) were used, respectively. The viability of the cells was assessed using a standard MTT protocol described elsewhere.^33^

The microbiological evaluation of the samples was performed using two representative bacterial strains: *Staphylococcus aureus* DSM 4910 and *Pseudomonas aeruginosa* DSM 22644. The bacterial strains were incubated at 37 °C in tryptic soy broth (TSB) (Becton Dickinson) for 18 h, harvested by centrifugation (4000 rpm, 3 min), and washed three times with PBS. The bacterial pellets were resuspended in Dulbecco’s PBS to obtain bacterial suspensions of 0.5 McFarland standard (10^8^ CFU/mL). The prepared bacterial suspensions were used in microbiological tests and incubated for 4 h with the polyurethane samples. Afterward, the samples were gently washed with PBS to remove all not-attached bacteria, following which they were stained using the LIVE/DEAD kit (BacLightTM Bacterial Viability Kit, Molecular Probes). The stained A549 cells and bacteria on the polyurethane samples were imaged using a BX63 fluorescent microscope (Olympus). The images were analyzed using the ImageJ 1.53k software.

### 2.4 Molecular Dynamics Simulation Details

MD simulations were conducted using the GROMACS 2021.5 software package.^34^ Force-field parameters for polyurethane, compatible with AMBER ff99SB-ILDN parametrization,^35^ were developed (see the Section 3). The parameters were derived starting with the ab initio geometry optimization of the fragments of the polyurethane polymer chains, namely, FRA, FRB, and FRC (vide infra), capped by the methyl groups. To this end, the density functional theory (DFT) method (B3LYP/cc-pVDZ) was employed in the Gaussian 09 package.^36^ Next, AmberTools^37^ were utilized to assign the atomic charges and the binding and nonbinding parameters, as well as transfer them to the GROMACS format. The same procedure was applied for generating force-field parameters for the oxidized fragments of polyurethane (Figure S1). Subsequently, the polyurethane force field was organized in the form of a monomer library, compatible with the pdb 2gmx tool from GROMACS. In this way, force-field parameters for polyurethane chains of different monomer numbers and oxidation could be easily derived and used in simulations. The developed force field is publicly available (https://github.com/tzvicky/polyu_ff).

Water molecules were described via the standard three-center TIP3P water model.^38^ Periodic boundary conditions were applied to the system with the particle mesh Ewald algorithm^39^ used to compute long-range electrostatic interactions. The Nose–Hoover thermostat^40^ and the Parrinello–Rahman barostat^41^ were employed to control the simulation temperature and pressure with time constants of 0.5 and 10 fs, respectively. The LINCS algorithm^42^ was used to constrain the bonds in the water molecules.

The typical investigated system consisted of ∼200,000 atoms; in total, over 25 μs of trajectories were collected, which were later analyzed using standard GROMACS tools. The initial packing of the polymer chains in the simulation box was realized using the Packmol software.^43^ The protocol for obtaining and equilibrating the initial structures of polyurethane was developed and is described in the Section 3. Overall, the equilibration procedure consisted of two steps: first, 10 replicas of pure polyurethane were simulated in the NVT ensemble at *T* = 600 K for 200 ns (to allow for rapid polymeric-chain reorganization) and subsequently in the NpT ensemble at *T* = 293 K and *p* = 1 atm.

## 3 Results and Discussion

### 3.1 Physicochemical Characterization of the Polyurethane Films

**Figure 1:**
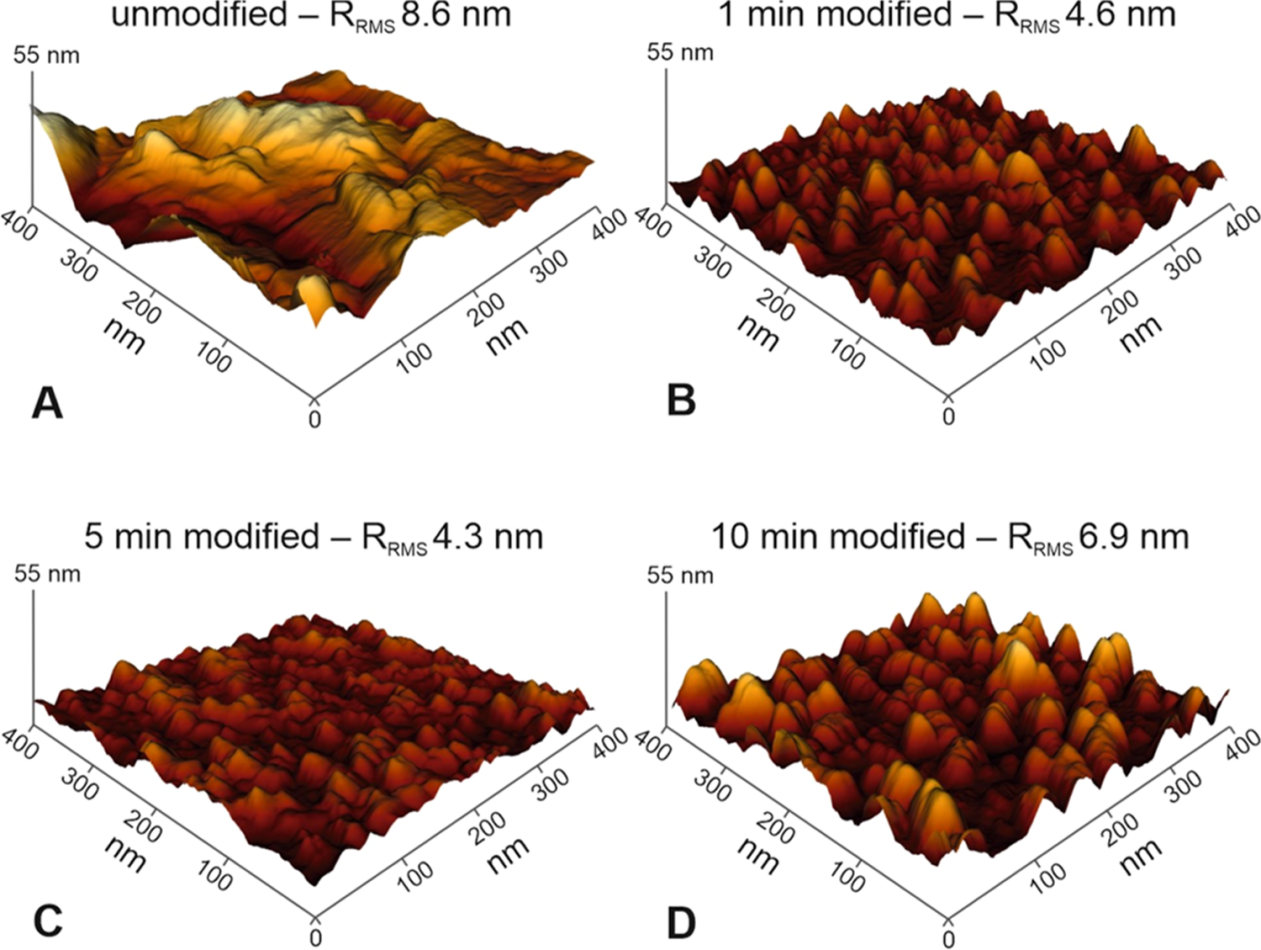
3D representation of the AFM surface topography of the unmodified (A) and plasma-modified polyurethane for 1 min (B), 5 min (C), and 10 min (D), accompanied by the R_RMS_ parameter values characterizing the changes in the surface roughness.

The surface properties of all the polyurethane samples, both unmodified and plasma-modified, were thoroughly characterized. The changes in the polyurethane surface topography upon treatment with oxygen plasma are presented in Figure 1. To quantify the differences between the modified samples, the sample roughness values, parametrized by the *R*_RMS_ of the polyurethane films, were compared.

The parent unmodified polyurethane foil is essentially planar with hills (bright regions) and valleys (dark regions) in the range of 50–100 nm. The O_2_ plasma treatment of polyurethane leads to changes in the surface topography; however, the corrugations are substantially less noticeable. Consequently, the roughness *R*_RMS_ of the polyurethane surface decreases significantly, although more surface irregularities can be observed in a lower range with a maximum amplitude in the *Z*-direction of up to 20 nm (see Figure 1A,B). Since polyurethane is composed of soft and hard segments exhibiting different susceptibilities to etching, nonuniform surface modification is expected upon contact with the plasma. As a result of amorphous domain’s preferential etching, empty space is created and allows the polymeric chains to reorganize.^44−47^ The slight *R*_RMS_ increase for the samples after long treatment times (Figure 1C,D) can be caused by such reorientation of the hard and soft segments and the formation of large domains.

**Figure 2:**
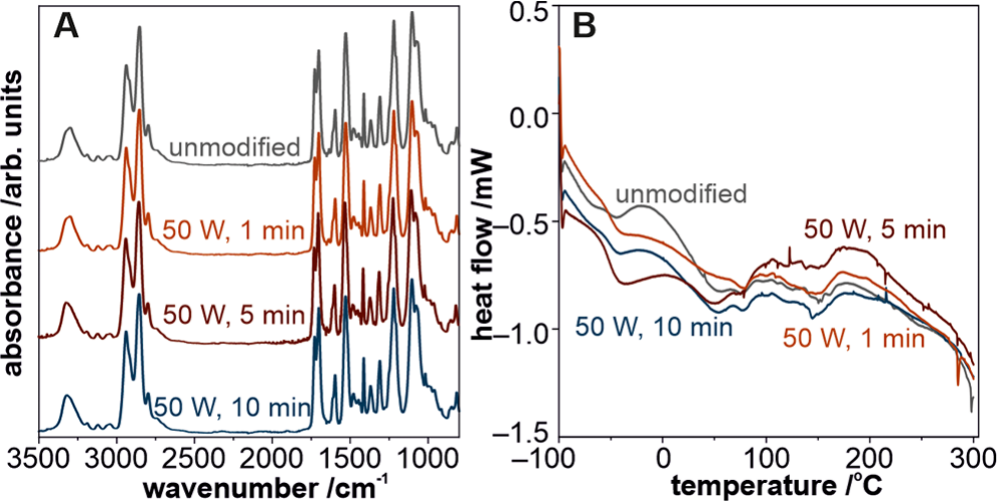
ATR-IR spectra (A) and the DSC curves (B) for the unmodified (black curve) and oxygen-plasma-modified polyurethane surfaces for 1 (red curve), 5 (brown curve), and 10 min (blue curve).

As previously reported,^48^ the main effect of the oxygen-plasma treatment lies in the surface functionalization via the generation of different oxygen-containing groups, such as −OH, −COOH, and −OOC. Such surface moieties were characterized by ATR-IR, XPS, and SIMS measurements. The ATR-IR spectra of the unmodified and oxygen-plasma-modified polyurethane films are shown in Figure 2A. The absorbance of typical IR bands in the high wavenumber region of the spectrum correlates with the urethane linkage (3300 cm^–1^, N–H stretching (hydrogen bonded) vibration; 3450 cm^–1^, non-hydrogen-bonded N–H bonds).^49^ The fingerprint region (between 1800 and 1000 cm^–1^) with several characteristics of the maxima for carbon–carbon, carbon–oxygen, oxygen–nitrogen, and carbon–nitrogen bonds are typical for the aromatic and aliphatic systems present in polyurethane chains.^50^ On comparing the spectra, no structural changes were observed after exposure to plasma with the applied parameters. To further substantiate this finding, DSC measurements were performed and their results are presented in Figure 2B. The profiles were collected in the temperature range of −100 to 300 °C, wherein all of the characteristic transformations of polyurethane structure occurred.^51^ All of the measured curves are virtually similar within the experimental errors, considering the inhomogeneity of the investigated polymeric material. These results complement the IR results, indicating that the structural integrity of the polyurethane chains was unchanged after plasma treatment.

**Figure 3:**
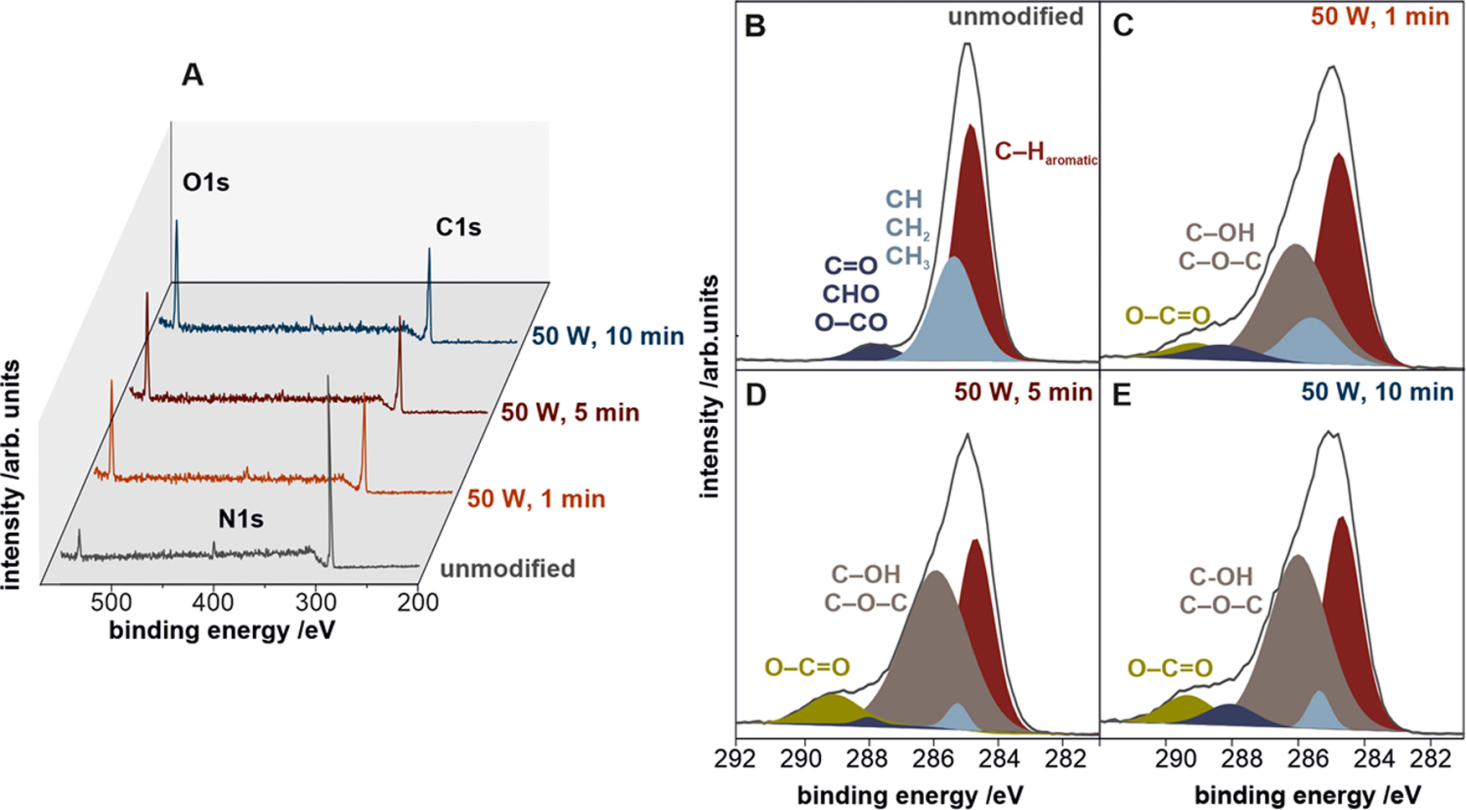
XPS survey scans (A) of the unmodified and oxygen-plasma-modified polyurethane samples together with the narrow scans for the C 1s for the unmodified (B) and oxygen-plasma-modified polyurethane samples for 1 (C), 5 (D) and 10 min (E). The raw XPS signal is represented as the black curve, and fitted peaks are in color.

To evaluate the composition of the polyurethane surfaces (before and after plasma treatment), XPS measurements were conducted. The survey spectra for the unmodified and modified surfaces for different plasma exposure times are presented in Figure 3A. Three main constituting elements are present at the polyurethane surface: carbon C 1s at 285 eV, nitrogen N 1s at 399 eV, and oxygen O 1s at 533 eV. A major increase in the intensity of the O 1s signal and a parallel decrease in the C 1s signal can be observed after the plasma treatment. The initial oxygen content of ∼9 at. % at the interfacial layer for the unmodified polyurethane increased to 22 at. % for the most modified sample, confirming the presence of oxygen-containing surface species. As a consequence, the carbon content changes from 89 to 75 at. %. A close inspection of the XPS spectra revealed a small contribution of nitrogen (present in the polyurethane chain); however, its concentration was near the detection limit and estimated to be below 4 at. %.

More in-depth insights into the chemical nature of the surface oxygen-containing groups can be inferred from the analysis of the C 1s signal. The high-resolution spectra (narrow scans) for carbon are presented in Figure 3B–E for the unmodified and oxygen-plasma-modified polyurethane surfaces. Various carbon components were fitted and attributed to specific chemical oxidation states. For the unmodified polyurethane sample, the C 1s XPS broad maximum (Figure 3B) consists of three components: the aromatic carbon at 284.8 eV, the aliphatic carbon at 285.3 eV, and the carbon–oxygen structures at 287.7 eV.

The increase in the intensity of the C 1s peak for the oxygen-plasma-modified samples is slightly more pronounced for long treatment times than for short times. Two new peaks appear in the range of 286.4–289.3 eV, attributed to the excessive oxidation of carbon to C–OH, C–O–C, and O–C = O groups, also confirmed by the chemical shift of O 1s (data not shown).^52,53^

**Figure 4:**
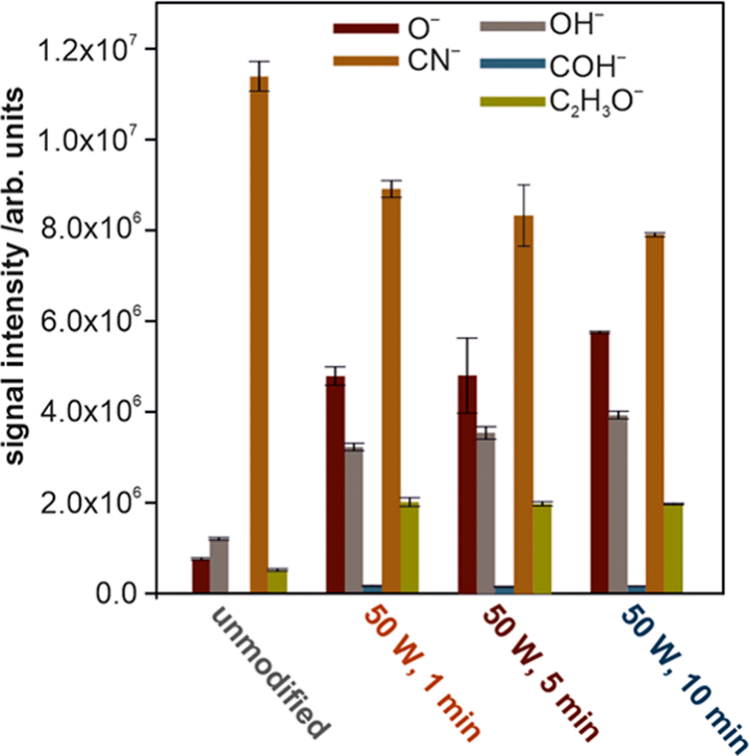
Comparison of the signal intensities of the surface oxygen-containing groups measured by SIMS for the unmodified and oxygen-plasma-modified polyurethane samples for 1, 5, and 10 min. Error bars represent the standard deviation.

For the complementary characterization of the surface modification, the static SIMS was employed. To monitor the surface modification, we focused on the peaks with characteristic masses of polyurethane chain fragments (O^–^, CN^–^, OH^–^, COH^–^, and C_2_H_3_O^–^) and extracted them from the raw data. The SIMS results for different modification parameters are presented in Figure 4. The most significant changes are related to an increase in the surface oxygen concentration. A high increase in the signal intensities for the negatively charged oxygen and oxygen-containing ions was observed from the unmodified to modified samples. For longer modification times, only slight changes in the surface oxygen-containing groups were observed. This trend for the oxygen content is in line with the XPS results described above. In contrast, a slight decrease in the amount of the CN^–^ surface ions can be noticed in SIMS, whereas in the XPS, the surface nitrogen content does not change upon plasma treatment. This can be explained by considering the different probing depths of these methods. As SIMS probes the surface within 1–2 topmost layers (corresponding to ∼1 nm of the sample), it reveals the rearrangement of the polymeric chains at the outermost surface.^54^

The presented results clearly show that although plasma treatment changes the surface topography, it does not affect the bulk structure of polyurethane. The changes indicated by XPS and SIMS are confined to the topmost surface region only. It may be thus concluded that exposure to plasma results in surface functionalization via the generation of oxygen-containing functional groups, whereas the superior bulk properties of the polymer are preserved (Figure 2). As expected, such a modification should result in dramatic changes in wettability and, therefore, the biologically relevant interactions at the polymer–cell interface.

**Figure 5:**
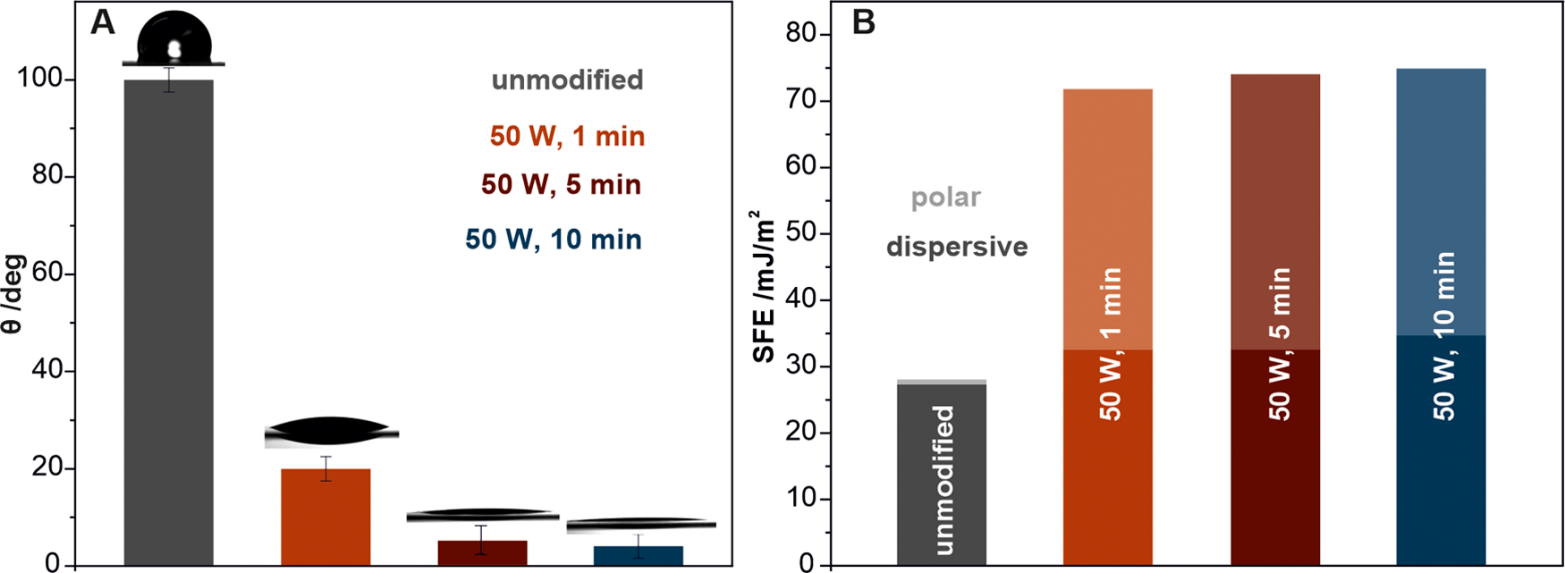
Water contact angles (A) for the unmodified (gray) and oxygen-plasma-modified polyurethane surfaces for 1 (orange), 5 (red), and 10 min (blue). The polar (light colors) and dispersive (dark colors) components of the SFE (B) were determined from the contact angle measurement. The error bars for the calculated SFE values are <5%.

The obtained XPS, SIMS, and AFM results revealed the modification of the surface chemistry (oxygen-containing functional groups) and topography (nano corrugations). Such induced changes strongly affect the polymeric surface hydrophilicity and, thus, the SFE. The first interaction of the biomaterial occurs at the polymer–water interface. Therefore, static contact angle measurements were performed to study the influence of the oxygen-plasma treatment on the wettability of the polyurethane surfaces. Since the wettability is a function of the coverage of the surface oxygen groups, it strongly depends on the applied plasma process parameters (Figure 5A). As expected, the initial contact angle of the untreated polyurethane of θ_w_ = 100° ± 3 rapidly decreased to θ_w_ = 25° ± 3 after 1 min of exposure to oxygen plasma. For longer times (over 5 min), no further decrease was observed and the surface is becoming totally hydrophilic with θ_w_ < 10°.

Based on the wettability changes, the Owens–Wendt method^32^ was implemented to determine the polyurethane SFE, as presented in Figure 5B. SFE is of primary importance when describing and correlating the surface properties with biological interactions in the body’s fluid environments (e.g., water, ions, sugars, proteins, and cells).^55,56^ The calculated SFE value for unmodified polyurethane was 27 mJ/m^2^, with the main contribution being from the dispersive component. After the oxygen-plasma treatment, the SFE value increased up to 75 mJ/m^2^ and the main observed changes are related to the dramatic increase in the polar component. This further affects the interactions with eukaryotic cells and bacteria.

### 3.2 Biological Characterization of Polyurethane

**Figure 6:**
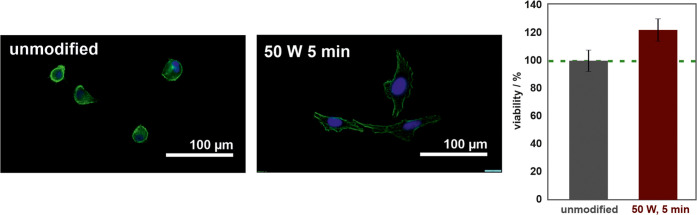
Images of the fluorescently stained A549 cells on the unmodified and oxygen-plasma-modified polyurethane (50 W, 5 min) surfaces, accompanied by the corresponding MTT viability results.

One of the major polyurethane applications is tubing for the respiratory system. Therefore, the A549 (adenocarcinomic human alveolar basal epithelial) cells were used as representatives in this study. The biological tests revealed a radical change in the cell morphology between the unmodified and oxygen-plasma-modified polyurethane samples. The representative images of the fluorescently stained A549 cells are shown in Figure 6 and indicate the significant influence of the oxygen-plasma treatment on the biocompatibility of polyurethane. The cells on the functionalized samples are well-spread and elongated, whereas those incubated on the unmodified polymer are round and exhibit motility. The MTT assay revealed that the oxygen-plasma treatment does not only influence the morphology of the cells but also increases the number of viable cells on the biomaterial surface by ∼20% (Figure 6). This beneficial effect of the oxygen-containing functional group generation is similar to those observed in other plasma-functionalized polymers^33,57^ and associated with the larger number of focal centers for cell adhesion.^58^

**Figure 7:**
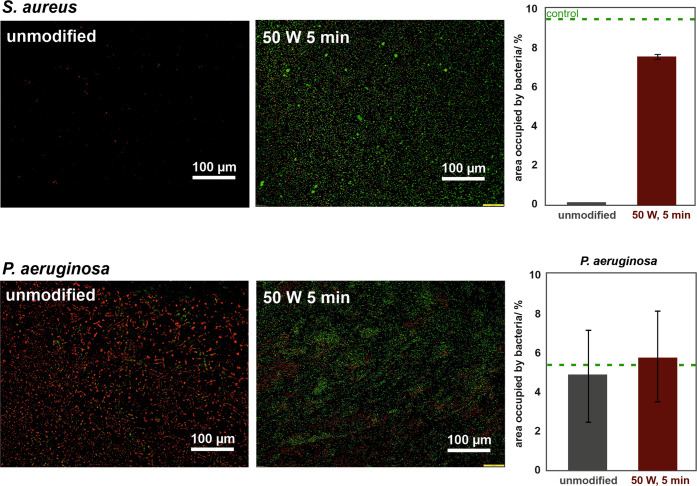
Microbiological test results (LIVE/DEAD staining, bacteria surface coverage) for *S. aureus* (upper panel) and *P. aeruginosa* (lower panel) on the unmodified and oxygen-plasma-modified polyurethane surfaces (green–live bacteria and red–dead bacteria).

Surface modification to increase biocompatibility is invaluable in developing novel biomaterials or improving already existing ones. However, considering the risk of BCIs, to fully evaluate biomaterials, microbiological tests should also be performed. Thus, the unmodified and modified (50 W, 5 min) polyurethane samples were tested against two bacteria strains that are most commonly responsible for after-implantation infections, i.e., *S. aureus* and *P. aeruginosa*. First, LIVE/DEAD staining was performed to identify the adhesion and bactericidal properties of the materials, followed by image analyses to assess the area occupied by the bacteria. The results are summarized in Figure 7.

The fluorescent images for the *S. aureus* strain (upper panel) revealed that the unmodified polyurethane surface is resistant to bacterial adsorption, as only a few single cells were observed. In contrast, the oxygen-plasma-modified surfaces significantly promoted the attachment of *S. aureus* (up to 8%) and its survival on the surface (the majority of the cells were stained green, indicating that the cells were alive bacteria cells). For *P. aeruginosa*, only dead, agglomerated bacteria cells were present on the unmodified polyurethane. After plasma treatment, the area occupied by the bacteria was ∼5%, which is comparable (within the experimental error) to that for the unmodified surface, although no bactericidal effect was observed. The larger error bars for *P. aeruginosa* are associated with the cell rod morphology and their high tendency to agglomerate on the investigated sample surfaces.

These results show the importance of a multifaceted approach in biomaterials science. Although the incorporation of oxygen-containing functional groups improves the surface biocompatibility for the A549 cell line, it creates a surface that is attractive for bacteria. Therefore, in this case, the plasma treatment significantly increases the risk of postimplantation infection. The results also highlight the necessity of controlling the surface state of implantable materials at the molecular level.

### 3.3 Computational Model of Polyurethane

**Figure 8:**
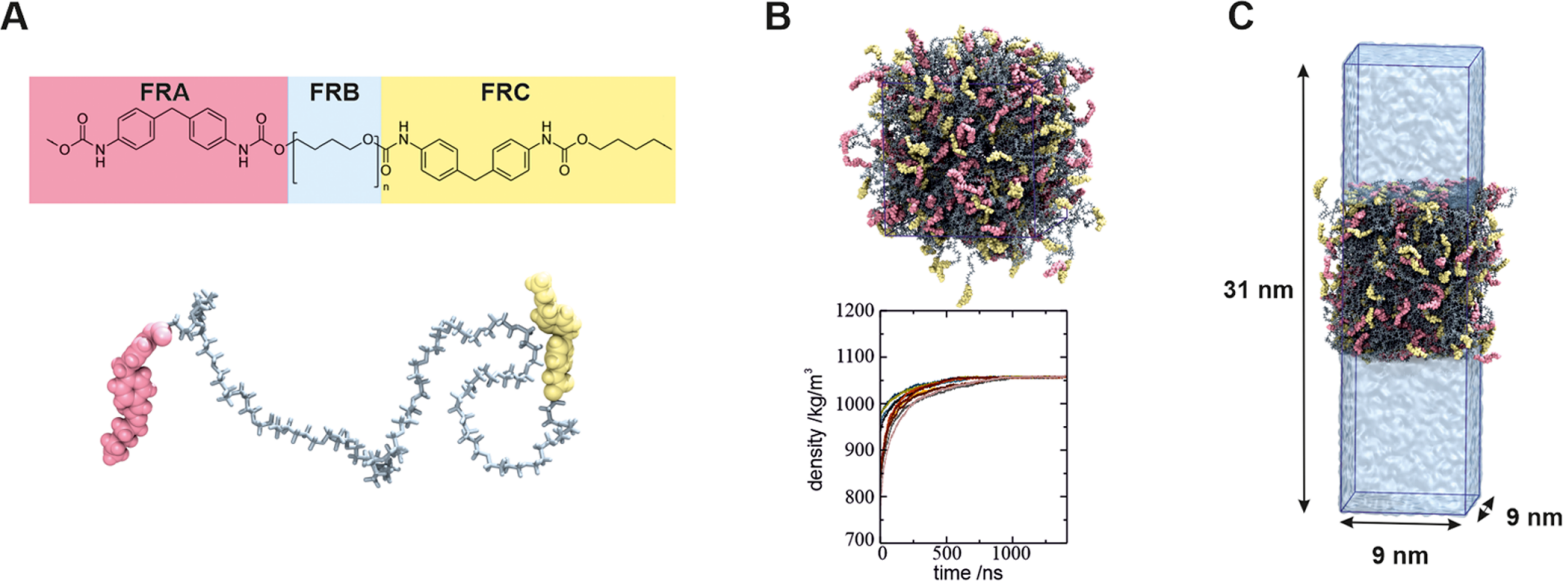
Chemical structure of the polyurethane chain used in this work (A). The simulation box of polyurethane and the mass densities of 10 independent MD runs (B). The MD snapshot of the polyurethane–water interface with the sizes of the initial simulation box (C). The color coding of the polyurethane structure is the same throughout the manuscript.

MD simulations were employed to gain atomistic insight into the biologically relevant interactions at the polyurethane surfaces. For the simulations, a new, fully atomistic force field of the polyurethane material was developed (see Methodology), with special care taken regarding its compatibility with existing computational models for biomolecules. Using this model, the fragments of the polymeric chain were generated and assembled to form the polyurethane chain with *n* = 27, as depicted in Figure 8A. The same was carried out for the oxidized versions of the polymer chain to simulate the oxygen-plasma-treated surfaces, as depicted in Figure S1.

In the MD simulations of the solid noncrystalline polymeric materials, obtaining a realistic initial three-dimensional molecular structure of the polymer is crucial, albeit practically challenging. The main problem lies in overcoming the multiple local conformational minima of the interacting polymer chain network. Here, this issue was addressed by elaborating an extensive computational protocol, including microsecond time scale MD simulations. To this end, 200 polyurethane chains were placed in a cubic simulation box with dimensions of 25 × 25 × 25 nm^3^ and equilibrated using a two-step NVT/NpT equilibration process (see the Methodology section). The equilibration runs lasted for 1500 ns with density values in all replicas converging after ∼800 ns of simulation, showing that relatively long equilibration is needed for such systems. The calculated density (1.06 g/cm^3^) was in good agreement with the experimental value (1.19 g/cm^3^), differing by ∼10%.

Using these equilibrated structures, slabs of polyurethane in water were created by solvating cubic boxes with water molecules (see Figure 8C) and performing another two-step NVT/NpT equilibration process. A similar procedure was conducted for the oxidized structures, except that in the first step, before the equilibration stage, oxidized polyurethane chains were created by replacing some of the original FRA and FRC fragments with their two oxidized variants (FRAox1 or FRAox2 and FRCox1 or FRCox2; see Figure S1). While the final composition of the oxidized structures was predetermined, the type of the variant substituting each fragment was chosen randomly.

### 3.4 Molecular Picture of the Polyurethane–Water Interface

**Figure 9:**
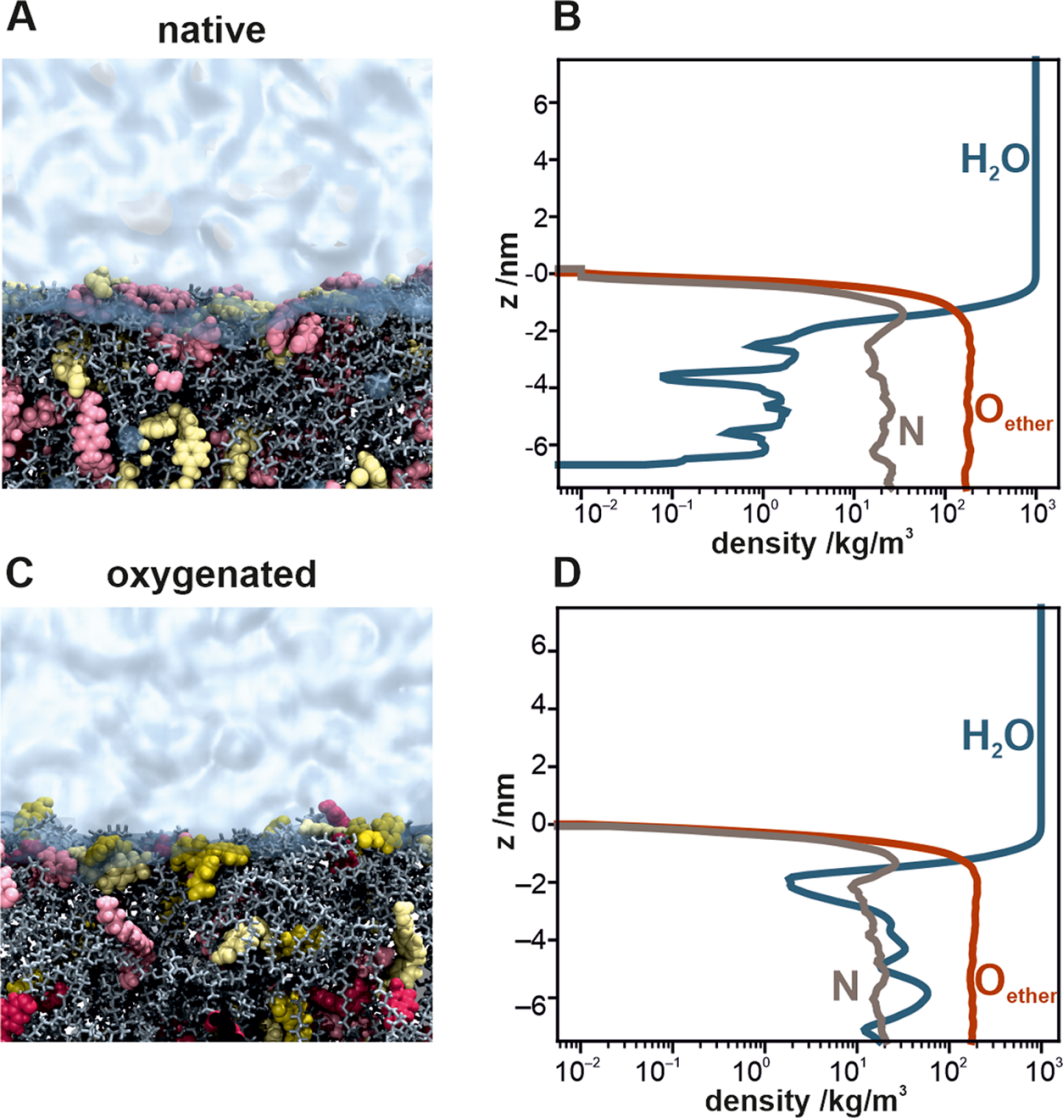
Representative MD snapshots of the unmodified and oxygenated polyurethanes ((A, C) respectively), accompanied by the mass density profiles of water (blue), ether oxygen from FRB (red), and nitrogen from both FRA and FRC (gray) at the unmodified (B) and modified (D) polyurethane–water interface. The oxidized FRA and FRC fragments are shown in dark red and dark yellow, respectively, in the MD snapshots. Zero on the *y*-axis denotes the polyurethane–water interface.

To analyze the polyurethane–water interface, the mass density profiles of water, nitrogen, and ether oxygen from FRB were calculated for the native (top row) and oxygenated (bottom row) polyurethane surfaces; the results are depicted in Figure 9. In both cases, the termini of the polymeric chains (containing the nitrogen) appear to be slightly enriched at the polymer–water interface, as compared with the case for the bulk polymer. Interestingly, water penetrates the bulk polyurethane to some extent. In MD simulations, water and polymer chains at the boundary are relatively dynamic, which agrees with the rearrangement behavior of hydrated polyurethane observed in earlier ESCA experiments.^59^

The water-penetration degree is 1 order of magnitude higher for the oxygenated polyurethane surface than for the unmodified surface. The aforementioned observations can be easily understood by considering the polarity of the different regions of the polyurethane chain. The terminals of the polymeric chains (FRA and FRC) are more polar than those of the poly(ether) fragments (FRB). This makes them prone to water exposure (cf. enrichment at the polymer–water interface). Additionally, the polar groups residing deep in the less-polar bulk polymer tend to attract water molecules to lower their nonfavorable interactions with less-polar environments (cf. water-molecule penetration of the bulk polyurethane). Further quantification of the specific interactions between water and the polymer groups (Figure S4) reveals that in the unoxidized polyurethane, where most of water–polymer contacts occur at the water-material boundary, FRC is predominantly responsible for interactions with water, followed by FRA and then the least polar FRB. In the oxidized polyurethane (Figure S5), water penetrates the material, interacting predominantly with the oxidized groups via their hydroxyl moieties.

Interestingly, oxygenation does not have any observable effect on the location of the polyether fragments (FRB) of the polymer, as evidenced by the virtually unchanged mass density profiles of the ether oxygen atoms. On the other hand, oxidation changes the overall profiles of oxygen at the polymer–water boundary (Figure S6). It should be noted that the molecular-level morphological changes of the polymer surface during hydration reported here occur over time- and size-scales accessible in MD simulations (hundreds of nanoseconds and tens of nanometers). Therefore, longer-term dynamics and larger-scale rearrangements cannot be captured. For instance, we cannot address the experimentally observed domains of ∼15 to 20 nm that were reported earlier using AFM.^60^ Nevertheless, MD simulations offer a valuable bridging link between the macroscopic observations and the molecular-scale interactions that underlie these phenomena.

**Figure 10:**
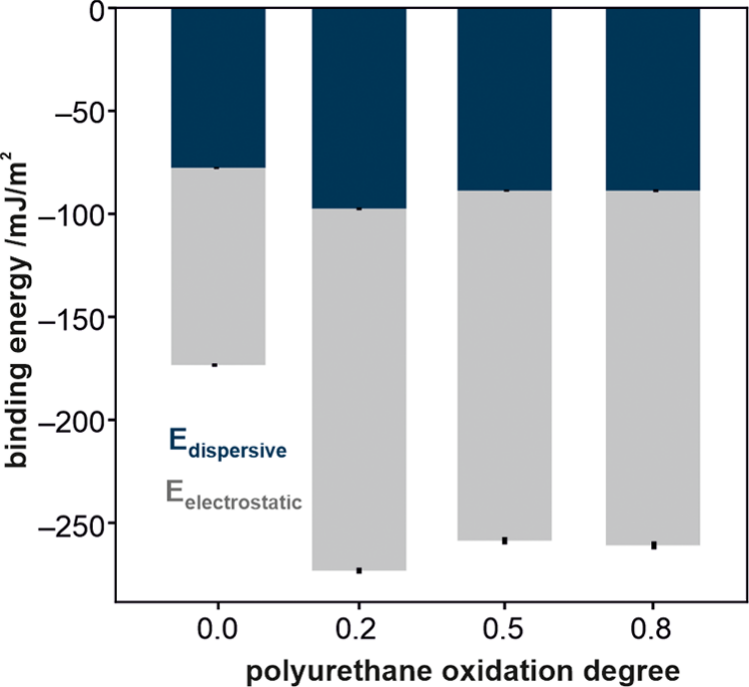
MD calculated water binding energies of the polyurethane surface with different degrees of oxidation degrees. The oxidation degree of zero corresponds to an unmodified polyurethane surface. The contributions from electrostatic (gray) and dispersive (blue) interactions are presented separately.

Figure 10 presents the calculated water binding energies for the polyurethane surface with different degrees of oxygenation. The binding energies were separated into electrostatic and dispersive components. The water binding energy increases from −173.3 to −273.2 mJ/m^2^ upon substitution of 20% of the ends of the polymeric chains by their oxidized variants. Interestingly, a further increase in the oxygen content in the polyurethane model exerted almost no effect on the binding energy. This content of oxygen corresponds to the experimentally obtained plasma-modified polyurethane surface and indicates the maximum saturation of the surface oxygen-containing groups (compare the data in Figures 5 and 10).

## 4 Conclusions

In this study, we successfully functionalized polyurethane films using oxygen plasma. By fine-tuning the plasma-treatment parameters, the modification was limited to the outermost surface without altering the bulk properties of the material. The oxygen-containing groups at the polymer surface improved its biocompatibility, as evidenced by the enhanced adhesion of human epithelial cells to the functionalized material surface. However, the observed augmented adhesion of bacteria suggests an increased risk of BCI. To supplement the experimental results with in-depth molecular descriptions, novel, fully atomistic models of polyurethane, unmodified and oxygenated, were devised and prepared using extensive MD simulations. Additionally, the interactions of water with the material were studied in detail. The simulations revealed that upon substitution of 20% of the ends of the polymeric chains by their oxidized variants, a further increase in the oxygen content in the polyurethane model exerted almost no effect on the water binding energy, revealing the surface saturation with oxygen-containing groups. The same trend was observed experimentally (SFE values) for the oxygen-plasma-treated surfaces (5 min, 50 W, and 0.14 mbar). Such state-of-the-art, combined experimental and theoretical approaches not only help to integrate our understanding of solid–water interfaces at the micro-, nano-, and atomistic-length scales but also are crucial for the knowledge-based development of novel biomaterials.

## Supporting Information Available

Additional molecular dynamics details for comparison of the developed in silico model with previous computational models of polyurethane available in the literature – Radial Distribution Functions and XRD, illustrating an amorphous structure of the polyurethane. Analysis of water interactions with individual groups of the polymer chains (PDF)
