# Supplementary material for: In Vitro and In Silico Studies of Functionalized Polyurethane Surfaces toward Understanding Biologically Relevant Interactions
Source: ACS Biomater Sci Eng. 2023 Nov 1;9(11):6112–22. doi: 10.1021/acsbiomaterials.3c01367 (PMC10646850; doi:10.1021/acsbiomaterials.3c01367)
Supplement: Supplementary file 1 — ab3c01367_si_001.pdf [file ab3c01367_si_001.pdf]

Supporting Information

In vitro and *in silico* studies of functionalised polyurethane surfaces towards understanding biologically relevant interactions

Paulina Chytrosz-Wrobel[1], Monika Golda-Cepa[1]*, Kamil Drozdz[2], Jakub Rysz[3], Piotr Kubisiak[1], Waldemar Kulig[4], Monika Brzychczy-Wloch[2], Lukasz Cwiklik[5]* and Andrzej Kotarba[1]

[1] Faculty of Chemistry, Jagiellonian University in Krakow, Gronostajowa 2, 30-387 Krakow, Poland,

[2]Department of Molecular Medical Microbiology, Chair of Microbiology, Faculty of Medicine, Jagiellonian University Medical College, Czysta 18, 31-121 Krakow, Poland

[3]Faculty of Physics Astronomy and Applied Computer Science, Jagiellonian University, Lojasiewicza 11, 30-348 Krakow, Poland

[4] Department of Physics, University of Helsinki, P.O. Box 64, FI-00014 Helsinki, Finland

[5]J. Heyrovský Institute of Physical Chemistry, Czech Academy of Sciences, Dolejškova 3, 18223 Prague, Czech Republic

* lukasz.cwiklik@jh-inst.cas.cz, mm.golda@uj.edu.pl

P. Chytrosz-Wrobel and M. Golda-Cepa equally contributed.

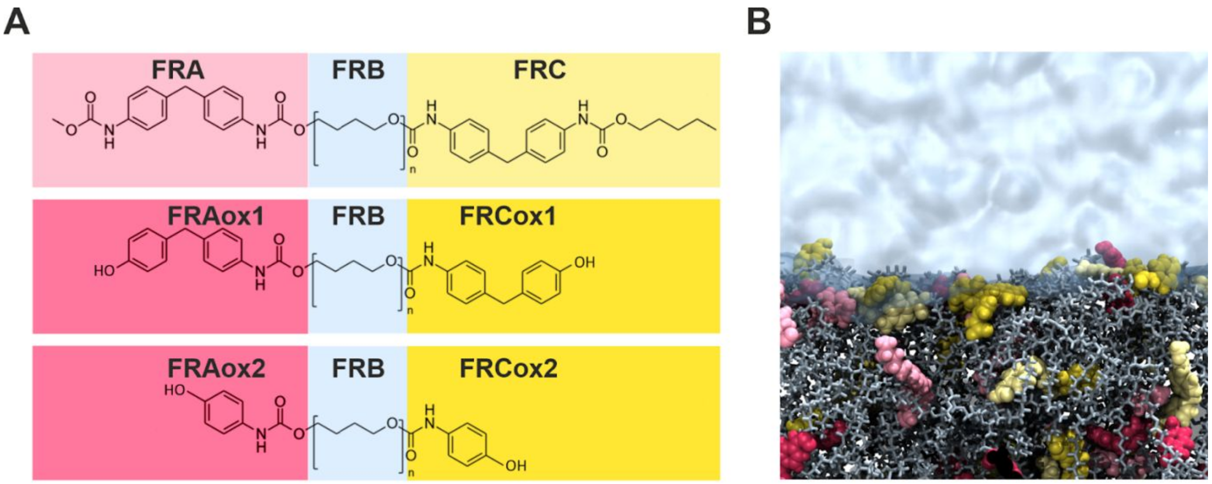

Fig.S1. Chemical structure of oxidized polyurethane chains used in this work.

Comparison of the developed *in silico* model with previous computational models of polyurethane

To compare the developed *in silico* polyurethane model with the models published previously [Ref. 24, 25], we analyzed the structural properties of bulk polyurethane obtained by utilizing our force field with those available in the literature. To this end, we calculated pair radial distribution functions (RDF) between the individual fragments in our model (FRA, FRB, FRC) and compared them with the previously published data. A direct comparison is complicated because of differences in definitions of groups along the polymer chain. Also, the simulated polyurethane materials have different characteristics (length of chains, initial conditions etc.)

The RDF calculated between the elastic FRB-FRB fragments (depicted in Fig.S2., blue) shows a well-pronounced peak at r=~0.5 nm with the value of ~2, followed by a less-pronounced peak at ~0.9 nm, and a weak peak at ~1.3 nm. Such behavior of RDF arises from the liquid-like structure of the chain fragments of the material, and similar behavior of a corresponding RDF was observed in Ref. 25. It also agrees with the disordered structure observed for our material via XRD (Fig.S3). The RDF calculated between the elastic and non-elastic fragments (FRA-FRB and FRC-FRB) shows no clear maxima, reporting on rather repulsive interactions between these fragments. Again, it demonstrates that the bulk of the simulated material is rather unstructured. Similar behavior for elastic-nonelastic fragments was observed in both previous models Ref. [24, 25]. In summary, the computationally-extensive model developed here shows overall agreement regarding structural data with previous computational models of polyurethane materials.

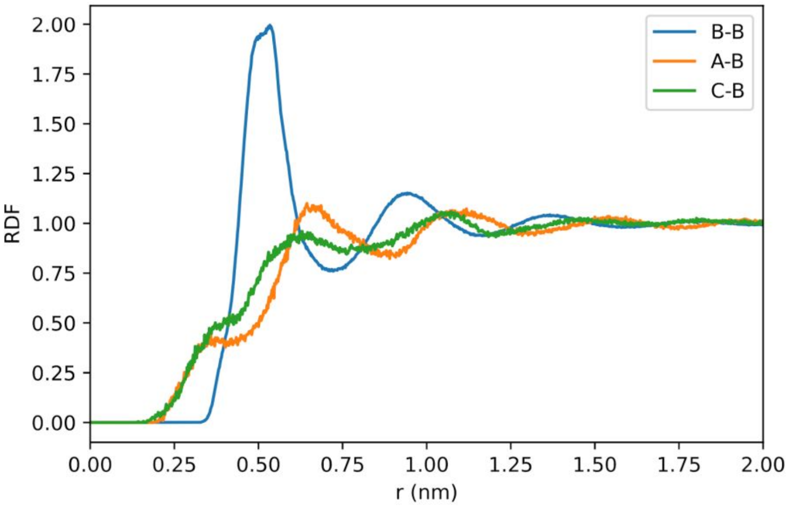

Fig. S2. Radial distribution functions for polyurethane fragments, FRA-FRB, FRB-FRB, FRC-FRB.

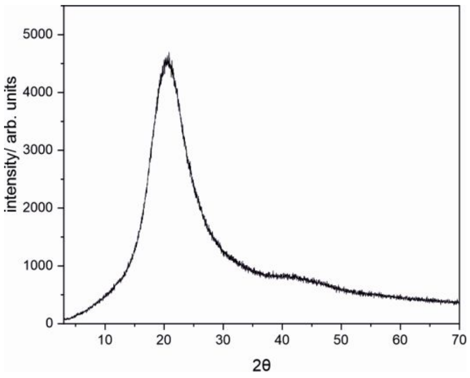

Fig. S3. The XRD results of studied polyurethane. The XRD data were collected on a Rigaku Miniflex System, Cu K**α** radiations at 10 mA and 10 kV, with the diffraction scanning rate of 1°/min.

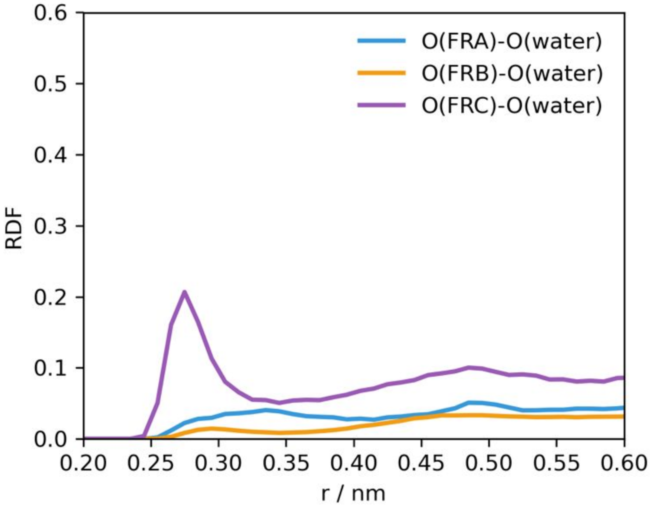

Fig. S4. Radial distribution functions calculated between oxygen atoms in the polyurethane fragments and the oxygen atom of water of the not oxidized system. Both interfacial and bulk groups are considered as most of water-unoxidized polymer contacts occur at the interface. The functions are not normalized to unity because of the asymmetry of the interface. The analysis demonstrates that most of water-unoxidized polymer contacts occur at FRC fragments.

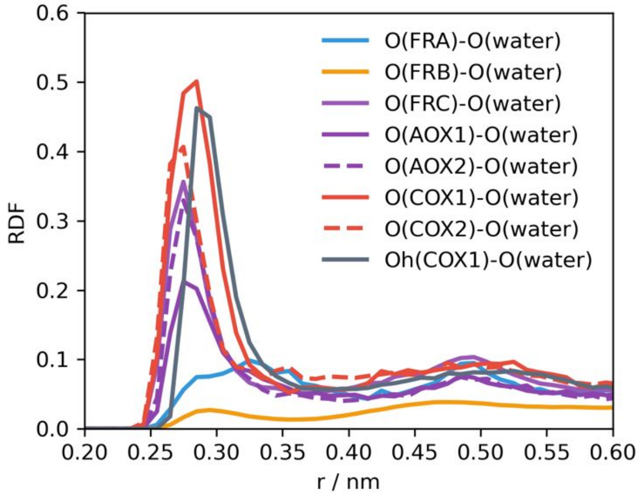

Fig. S5. Radial distribution functions calculated between oxygen atoms in the polyurethane fragments and the oxygen atom of water of the oxidized system. For COX1, also the function for hydroxyl oxygen (Oh)-water oxygen is shown to demonstrate that most of water-oxidized polymer interactions occur between hydroxyl groups of oxidized fragments and water. Only sub-interfacial groups (>1 nm from the interface) are considered to analyze the penetration of the oxidized polymer by water. The functions are not normalized to unity because of the asymmetry of the interface.

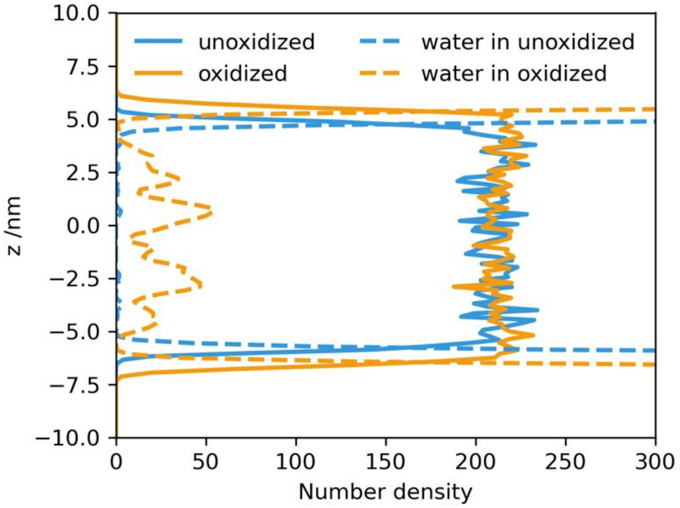

Fig. S6. Number density (atoms per nm^3) profiles of oxygen atoms in the polyurethane structure and water oxygen calculated for unoxidized and oxidized systems. For the polymer, full profiles along the MD simulation box are shown with zero at the y-axis denoting the midplane of the polymer placed in the middle of the simulation box. Oxidation changes the distribution of oxygen at the boundaries. In the sub-interfacial region, the oxygen density does not significantly change after oxidation because an increase due to oxidation is compensated by the reduced overall number of oxygen atoms in the truncated oxidized fragments.
